# Warburg effect‐related risk scoring model to assess clinical significance and immunity characteristics of glioblastoma

**DOI:** 10.1002/cam4.6627

**Published:** 2023-10-21

**Authors:** Rong Zhang, Can Wang, Xiaohong Zheng, Shenglan Li, Weichunbai Zhang, Zhuang Kang, Shuo Yin, Jinyi Chen, Feng Chen, Wenbin Li

**Affiliations:** ^1^ Department of Neuro‐Oncology, Cancer Center, Beijing Tiantan Hospital Capital Medical University Beijing China

**Keywords:** drug sensitivity, glioblastoma, immune microenvironment, prognostic model, Warburg effect

## Abstract

**Background:**

Glioblastoma (GBM), the most common primary malignant brain tumor, has a poor prognosis, with a median survival of only 14.6 months. The Warburg effect is an abnormal energy metabolism, which is the main cause of the acidic tumor microenvironment. This study explored the role of the Warburg effect in the prognosis and immune microenvironment of GBM.

**Methods:**

A prognostic risk score model of Warburg effect‐related genes (Warburg effect signature) was constructed using GBM cohort data from The Cancer Genome Atlas. Cox analysis was performed to identify independent prognostic factors. Next, the nomogram was built to predict the prognosis for GBM patients. Finally, the drug sensitivity analysis was utilized to find the drugs that specifically target Warburg effect‐related genes.

**Results:**

Age, radiotherapy, chemotherapy, and WRGs score were confirmed as independent prognostic factors for GBM by Cox analyses. The C‐index (0.633 for the training set and 0.696 for the validation set) and area under curve (>0.7) indicated that the nomogram exhibited excellent performance. The calibration curve also indicates excellent consistency of the nomogram between predictions and actual observations. In addition, immune microenvironment analysis revealed that patients with high WRGs scores had high immunosuppressive scores, a high abundance of immunosuppressive cells, and a low response to immunotherapy. The Cell Counting Kit‐8 assays showed that the drugs targeting Warburg effect‐related genes could inhibit the GBM cells growth in vitro.

**Conclusion:**

Our research showed that the Warburg effect is connected with the prognosis and immune microenvironment of GBM. Therefore, targeting Warburg effect‐related genes may provide novel therapeutic options.

## Graphical abstract

In this study, a reliable Warburg effect‐related genes risk scoring model was identified. This model has a significantly excellent predictive value for the prognosis of glioblastoma (GBM) patients, suggests their immunotherapy response, and identifies new GBM therapeutic targets.

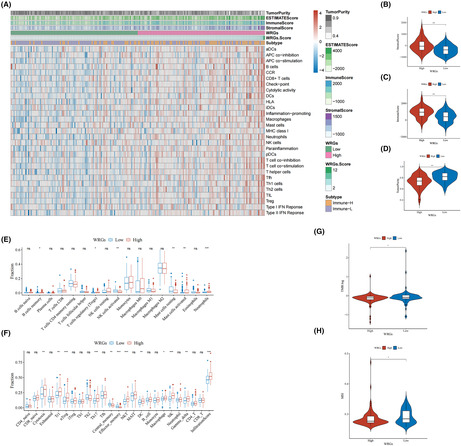

## 1 INTRODUCTION

Glioblastoma (GBM) is the most prevalent primary malignant brain tumor, accounting for 48.6% of all malignant central nervous system (CNS) tumors.^1^, ^2^ It has a poor prognosis, with only 7% of patients surviving 5 years after diagnosis. After receiving standard treatment, patients with GBM had a 14.6‐month median overall survival (OS).^3^ Many studies have been conducted on the factors that could affect the prognosis of GBM, such as age, radiotherapy, chemotherapy, and O^6^‐methylguanine‐DNA methyltransferase (MGMT) promoter.^3^, ^4^ However, the complex pathogenesis and molecular heterogeneity of GBM render it difficult for the current prognostic factors to explain the progression of the disease, necessitating the investigation of other prognostic factors.

The Warburg effect, first observed by Otto Warburg in the 1920s, is a phenomenon of abnormal glucose metabolism in cancer.^5^ It refers to the fact that tumor cells participate in aerobic glycolysis in the presence of enough oxygen, subsequently leading to the production of a significant amount of lactic acid.^6^, ^7^ Lactic acid has a significant effect on several carcinogenesis processes, involving metastasis, angiogenesis, metabolism, and immunosuppression.^8^ Recent research has demonstrated that elevated lactic acid produced by Warburg effect is a poor prognostic indicator for metastatic lung cancer.^9^ The effect of lactate has also been established and confirmed in the prognosis of colorectal cancer,^10^ lung adenocarcinoma,^11^ and esophageal squamous cell carcinoma.^12^ Moreover, recent research has demonstrated that Warburg effect‐related genes have a significant effect on tumor progression and prognosis. DExD‐box helicase 39B (DDX39B) can promote colorectal cancer metastasis through activating the Warburg effect, which was confirmed as a poor prognostic indicator for the prognosis of colorectal cancer.^13^ Basic leucine zipper and W2 domain‐containing protein 1 contributed to the growth and poor prognosis of pancreatic ductal adenocarcinoma by promoting the Warburg effect.^14^ Previous studies have also shown that Warburg effect‐related genes such as monocarboxylate transporter 1,^15^ Glucose transporter‐1,^16^ and SoLute Carrier family 9A1^17^ have a negative effect on the prognosis of GBM. However, a comprehensive Warburg effect signature for the prediction of the prognosis of GBM has not been established to date.

In addition, high lactate levels produced by the Warburg effect have been demonstrated as a key immunosuppressive metabolite in the tumor microenvironment (TME).^18^ Research has shown that the Warburg effect leads to the formation of an immunosuppressive microenvironment by maintaining a low potential hydrogen value in the TME,^18^, ^19^ such as by polarizing tumor‐associated macrophages to favor the M2 phenotype,^20^, ^21^ promoting the depletion of cytotoxic T cells,^22^ and thereby promoting tumor progression. However, the role of the Warburg effect on other cells involved in tumor immunity has not been clearly reported, especially the role of the Warburg effect on the immune cell profile in the GBM immune microenvironment. Given that previous studies have reported the existence of severe immunosuppression in GBM,^23^ elucidating the above issues may provide novel approaches for immunotherapy of GBM. Therefore, the role of the Warburg effect on the immune microenvironment of GBM is worthy of further exploration.

Recent research has demonstrated that the Warburg effect alters the tumor microenvironment and promotes angiogenesis, immunosuppression, formation of tumor‐associated fibroblasts, and drug resistance.^24^ Therefore, targeting Warburg effect‐related genes would be a promising approach for treating GBM. Several preclinical and early‐stage clinical studies have demonstrated that targeting the Warburg effect is effective in inhibiting tumor progression. For example, previous research has shown glycolysis as a therapeutic target in colorectal cancer, and inhibitors of glycolysis have been used in clinical trials.^24^ Another study investigated the relationship between the Warburg effect and glioma and proposed that the ketogenic diet might be effective in the therapy of low‐grade glioma (LGG) by affecting the Warburg effect.^25^ Inhibition of Aurora kinase A has been shown to affect the cellular metabolism of GBM by the reversal of the Warburg effect.^26^ Nevertheless, there are currently no drugs available that specifically target the Warburg effect‐related genes in the clinical treatment of GBM.

Although several prognostic models for GBM have been built in the past, a more recent and accurate prognostic model is also necessary given the new diagnostic criteria for GBM in the fifth edition of the World Health Organization (WHO) Classification of Tumors of the CNS in 2021.^27^

In this research, we investigated the role of the Warburg effect on the prognosis and immune microenvironment of GBM patients using bioinformatics techniques. Moreover, we also explored the drugs that target Warburg effect‐related genes and validated them utilizing Cell Counting Kit‐8 (CCK‐8) assays. This study gives a novel tool for the clinical prediction of prognosis in GBM and contributes to the development of new therapeutic strategies.

## 2 MATERIALS AND METHODS

### 2.1 Sources of datasets and pretreatments for GBM

First, the RNA sequencing (RNA‐seq) data, somatic cell datasets, and clinical data for all glioma patients were acquired using The Cancer Genome Atlas (TCGA) database. Next, telomerase reverse transcriptase (*TERT*) promoter mutation, epidermal growth factor receptor (*EGFR*) gene amplification, or +7/−10 copy number changes in isocitrate dehydrogenase (IDH)‐wildtype diffuse astrocytomas and IDH‐wildtype GBM were selected as the training set based on the WHO CNS5. Duplicate patient records were deleted. Lastly, data from defined GBM patients based on WHO CNS5 were included for further analysis. Single‐nucleotide polymorphism (SNP) and copy number variation (CNV) data for each GBM patient were retrieved from the University of California Santa Cruz Xena website. The validation set for this study was obtained after excluding LGG and IDH mutant GBM from the Chinese Glioma Genome Atlas (CGGA) database. The data on gene expression from the training and validation cohorts were log2 transformed [log2 (FPKM+1)]. Fragments per kilobase million (FPKM) data were utilized in single‐sample gene set enrichment analysis (ssGSEA) analysis. Transcripts per kilobase million (TPM) data was used for CIBERSORTx and ImmuCellAI analysis. GSE16011 cohort from the Gene Expression Omnibus (https://www.ncbi.nlm.nih.gov/geo/) database was downloaded and used to further validate the risk score model. The P27 cell line was derived from GBM patients in the Department of Neuro‐oncology, Cancer Center, Beijing Tiantan Hospital, and was sought after ethical approval (IRB: KY 2021‐153‐03) and patient consent. The cells were handled according to the protocols approved by the ethical committee of Beijing Tiantan Hospital, Capital Medical University. U87, U251, and LN229 GBM cell lines were donated by Beijing Tiantan Hospital Affiliated to Capital Medical University. The genetic types of U87, U251, and LN229 were as follows: U87, U251, and LN229 were IDH1/2‐wildtype cell lines with CDKN2A/B homozygous deletions. EGFR gene amplification in U251 and LN229 cell lines, but not in U87 cells; phosphatase and tensin homolog (PTEN) gene mutant in U87 and U251 cells; PTEN gene wildtype in LN229 cell line; tumor protein p53 (TP53) gene mutant in LN229 and U251 cells; TP53 gene wildtype in U87 cells.

### 2.2 Definition of Warburg effect‐related genes

Genes associated with the Warburg effect were identified from previous studies (Table S1). The criteria for inclusion of Warburg effect‐related genes were as follows: (1) genes identified to be directly involved in the Warburg effect and (2) genes directly related to the Warburg effect rather than all genes in a same signal pathway.

### 2.3 Mutation analysis of Warburg effect‐related genes

The locations of the Warburg effect‐related genes were mapped on 46 chromosomes using CNV data. The mutation frequency of Warburg effect‐related genes and oncoplot waterfall plots were constructed using SNP data.

### 2.4 Constructing and validating a prognostic risk score model for Warburg effect‐related genes (Warburg effect signature)

By Kaplan–Meier (K‐M) survival analysis, the prognostic value of Warburg effect‐related genes for GBM was evaluated, and genes with prognostic significance were used for further analysis. Based on these prognostic genes, we performed univariate COX regression analysis and least absolute shrinkage and selection operator (LASSO) regression (R package “glmnet” and “MASS”) to gain the Warburg effect signature for predicting the prognosis of GBM. Eight genes, G Protein‐Coupled Receptor 68 (GPR68), Mitochondrial Pyruvate Carrier 1 (MPC1), Solute Carrier Family 16 Member 1 (SLC16A1), Signal Transducer And Activator Of Transcription 3 (STAT3), Transketolase Like 1 (TKTL1), Ribonucleotide Reductase Regulatory Subunit M2 (RRM2), Mechanistic Target Of Rapamycin Kinase (MTOR), and Toll Like Receptor 4 (TLR4), were obtained by appropriate λ value (representing the appropriate number of genes in the model) and the least Akaike information criterion (AIC) value (representing the optimal choice of the model) to construct the Warburg effect signature. The expression of the Warburg effect signature in GBM patients was defined as the Warburg effect‐related genes (WRGs) score. The following formula was employed for calculating the WRGs score: WRGs score = $\sum_{i=1}^{n}\text{Coef}\left(i\right)\times x\left(i\right)$, where coef (*i*) and *x* (*i*) represent the regression coefficients of the multivariate Cox regression model and the expression of Warburg effect‐related genes, respectively.^28^ A risk score of Warburg effect signature for per GBM patient was then calculated. Based on the cutoff value, all TCGA GBM patients were categorized into high‐WRGs scores (high‐WRGs) and low‐WRGs scores (low‐WRGs) groups. The performance of the risk model was then assessed by comparing the survival rates of two groups using the K‐M curve. Lastly, the risk model developed from the TCGA GBM cohort was tested in the CGGA GBM cohort using the same approach.

### 2.5 Independent prognostic value of the Warburg effect signature

The WRGs score and clinical information from GBM patients were integrated for univariate and multivariate Cox regression model analysis. The forest plot was created with the “forestplot” package of R, showing the *p*‐value, hazard ratio, and 95% confidence interval of each parameter.

### 2.6 Nomogram construction

Independent characteristics were chosen by multivariate Cox stepwise regression analysis in the TCGA GBM set to build a nomogram to predict the prognosis of GBM patients at 6, 12, and 24 months. The reliability of nomogram prediction was statistically evaluated using the calibration curve, time‐dependent receiver operating characteristic (ROC) curves, concordance index (C‐index), and Brier Score. The nomogram was finally validated using the aforementioned methods in the CGGA validation set.

### 2.7 Analysis of immune infiltration, tumor mutation burden (TMB), and microsatellite instability (MSI)

The gene scores of 29 gene sets associated with immunity were evaluated in each sample using the ssGSEA. By comparing differences between 29 immune‐related pathways, patients were categorized into clusters with high and low immune responses. The tumor purity and TME scores (including the ESTIMATE score, Stromal score, and Immune score) were analyzed by The Estimation of Stromal and Immune cells in Malignant Tumor tissues using Expression data (ESTIMATE). The heatmap and WRGs score together showed the degree of immune infiltration. The percentages of immune cells were calculated using CIBERSORTx and ImmuCellAI. The role of WRGs score on GBM immunotherapy responses was shown using violin plots to show MSI and TMB in the two groups, respectively.

### 2.8 Drug‐sensitivity analysis based on the Warburg effect signature

Using data on drug response from the Cancer Therapeutic Response Portal (CTRP) and the Cancer Cell Lines Encyclopedia (CCLE), drugs associated with the Warburg effect signature were investigated using Spearman's correlation analysis. The effect of different concentrations of inhibition of arginase with (S)‐(2‐boronoethyl)‐L‐cysteine (BEC [catalog number: S7929, Selleck.cn]) ranging from 0 to 1000 μmol/L on the viability of U87, U251, LN229 GBM, and P27 cell lines for 72 h was studied using CCK‐8 assays. The effect of BEC on glucose uptake and lactate generation was studied between BEC‐treated and untreated GBM cell lines. BEC‐treated referred to BEC treated with 100 μM BEC. U87, U251, LN229, and P27 GBM cell lines were divided into treated‐BEC groups and nontreated‐BEC groups. After 48 h of culture, the culture supernatant was collected. Then, the cell viability of two groups was detected by CCK‐8, respectively. Next, the glucose and lactate contents in the culture supernatant were quantitatively analyzed by a glucose detection kit (Nanjing Jiancheng Bioengineering Institute, A154‐2‐1) and a lactate detection kit (Nanjing Jiancheng Bioengineering Institute, A019‐2‐1), respectively. Finally, glucose uptake and lactate generation were calculated for each unit of cell viability. The glucose consumption and lactate generation experiments were repeated five times (*n* = 5), respectively.

### 2.9 Cellular culture

U87, U251, LN229, and P27 GBM cell lines were cultured in DMEM High Glucose (EallBio, 03.1006C) complete media with 10% FBS (ScienCell, 0500, San Diego, CA, USA), 1% penicillin–streptomycin (Gibco, 15140‐122, NY, USA), and 2% prophylactic Plasmocin™ (Invivogen, ANT‐MPP, CA, USA). Once every 3 days, the cells were passaged. Every 2 days, the medium was changed.^29^

### 2.10 Statistical analysis

The R software (version 4.1.1) and Bioconductor package were utilized for all bioinformatics data analysis. The Graphpad prism was used for laboratory data analysis. Spearman's correlation coefficient |R| > 0.3 and a *p*‐value of <0.05 were considered statistically significant.

## 3 RESULTS

### 3.1 Genomic characteristics of Warburg effect‐related genes

**FIGURE 1:**
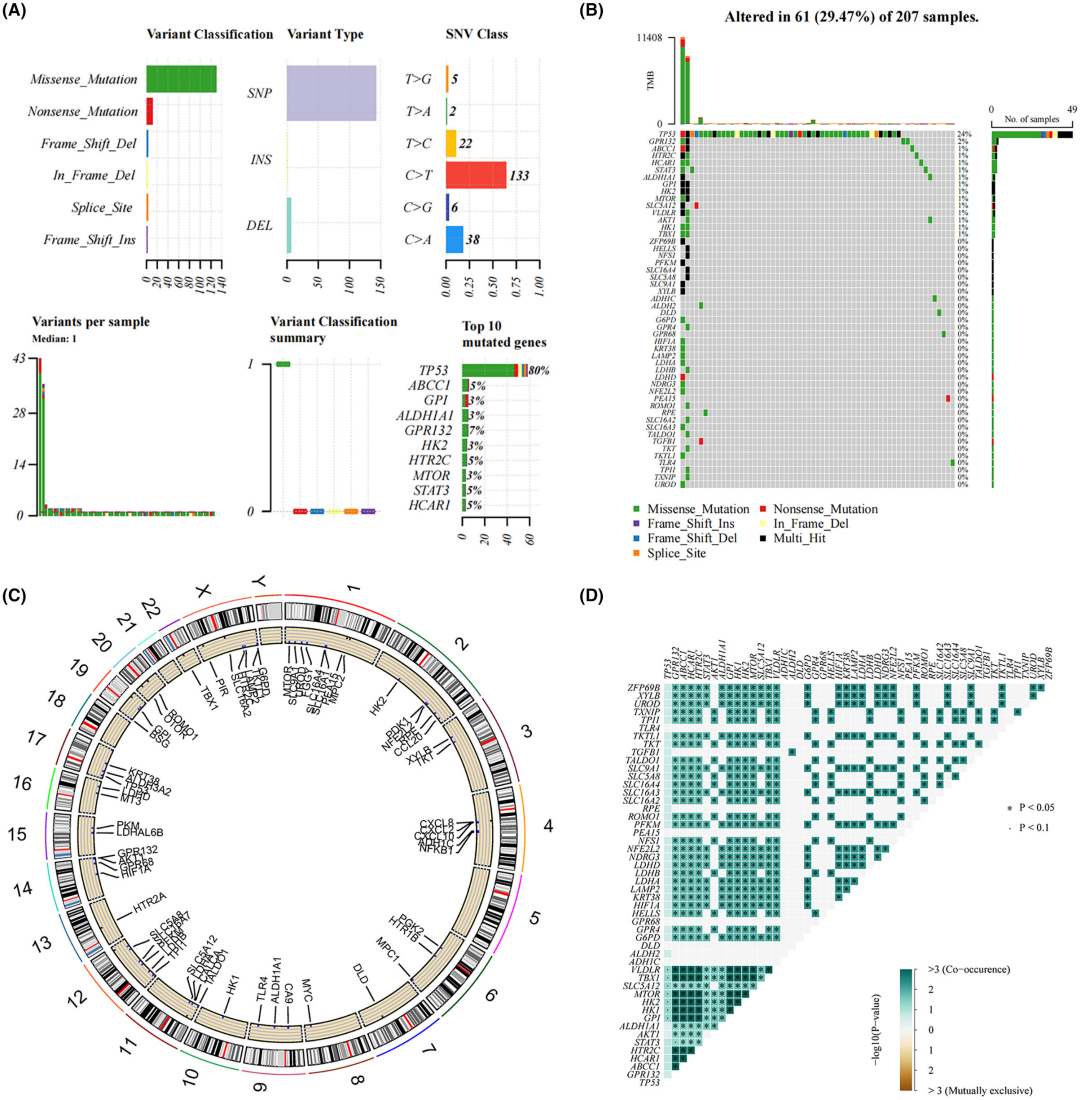
Genomic features. (A) Somatic mutations of Warburg effect‐related genes. (B) List of the most frequently altered Warburg effect‐related genes. (C) The location of Warburg effect‐related genes on chromosomes. (D) Correlation of Warburg effect‐related genes. The depth of the colors indicates the strength of the relevance. *p < 0.05.

Using the TCGA GBM cohort, somatic mutation analysis and CNV were carried out to investigate genetic characteristics. Variant classification, variant type, gene mutation proportion, and single‐nucleotide variant (SNV) were all assessed in the somatic mutation analysis (Figure 1A). Of the SNVs that were found, C>T mutations were the most prevalent. Somatic mutation analysis revealed the percentages of mutations in TP53 (80%), GPR132 (7%), HCAR1 (5%), HTR2C (5%), ABCC1 (5%), STAT3 (5%), MTOR (3%), GPI (3%), ALDH1A1 (3%), and HK2 (3%). Figure 1B showed the alterations with Warburg effect‐related genes in 61 GBM patients in the TCGA training set, where missense mutations were the predominant type of genetic alterations. CNV analysis revealed the location of Warburg effect‐related genes on the chromosomes. According to the results, chromosome 1 contained the greatest amount of CNV variants in the WRG, followed by chromosome X, chromosome 2, chromosome 4, and chromosome 12 (Figure 1C). A correlation between Warburg effect‐related genes in GBM was found by Co‐mutation correlation analysis (Figure 1D). Therefore, we preferred to construct a gene set rather than a single Warburg effect‐related genes to investigate the role of the Warburg effect on the prognosis and immune microenvironment of GBM.

### 3.2 Construction of Warburg effect signature

**FIGURE 2:**
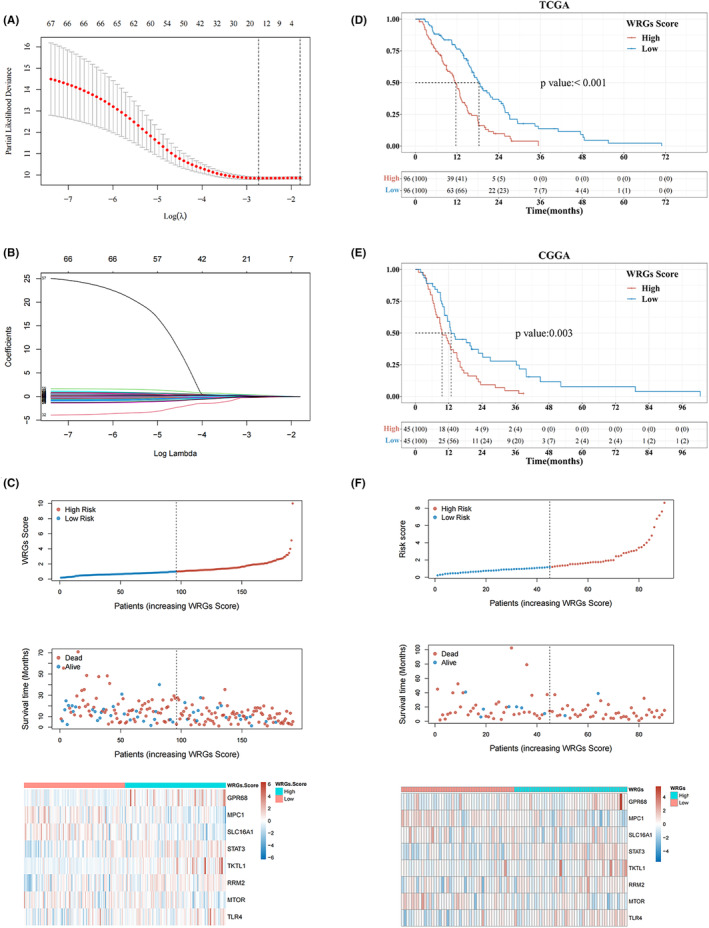
Construction of the prognostic risk model in the TCGA cohort and its validation in the CGGA cohort. (A and B) Eight Warburg effect‐related genes were selected using LASSO analysis with the appropriate λ value and the least Akaike information criterion (AIC) value. (C) Distribution of the survival risk, survival status, and expression of eight Warburg effect‐related genes between the high‐WRGs score (high‐WRGs) and low‐WRGs score (low‐WRGs) groups in the training cohort. (D and E) Comparisons of the overall survival between the high‐ and low‐WRGs groups in the TCGA and CGGA cohorts. (F) Distribution of the survival risk, survival status, and expression of eight Warburg effect‐related genes between the low‐ and high‐WRGs groups in the validation cohort. The depth of the colors represents the strength of the correlation. Red represents a high expression, and blue represents a low expression.

The Warburg effect signature for the prediction of the prognosis of GBM was developed by variable selection and model construction using LASSO Cox regression analysis (Figure 2A,B). GPR68, MPC1, SLC16A1, STAT3, TKTL1, RRM2, MTOR, and TLR4 were obtained to construct the risk score model. Based on the expression of each gene in the Warburg effect signature, we accessed the risk score for each patient. The formula was as follows: WRGs score = (0.5125) * GPR68 expression level + (−0.2607) * MPC1 expression level + (−0.5221) *SLC16A1 expression level + (0.7147) * STAT3 expression level + (0.2894) * TKTL1 expression level + (0.2817) * RRM2 expression level + (−0.5515) * MTOR expression level + (0.2093) * TLR4 expression level. The prognostic WRGs score was then determined for every patient in the GBM cohort of the TCGA. Next, GBM patients were categorized into high‐WRGs score (high‐WRGs) and low‐WRGs score (low‐WRGs) groups based on the median (0.62) as the cutoff value. The risk plot illustrated survival risk, survival status, and Warburg effect signature expression levels in the two groups (Figure 2C), which revealed that survival time shortened as the WRGs score increased. High expression of GPR68, STAT3, TKTL1, RRM2, and TLR4 suggested a poor prognosis for GBM patients, while high expression of MPC1, SLC16A1, and MTOR indicated a long OS for patients. Additionally, the K‐M survival curve illustrated that the prognosis of the high‐WRGs group was considerably poorer than the low‐WRGs group (*p* < 0.001) (Figure 2D). We performed external validation using the CGGA set to confirm the prediction value of this signature. The survival curve demonstrated that the prognosis for the high‐WRGs group was much poorer as compared to the low‐WRGs group (*p* = 0.003) (Figure 2E). The risk plot also revealed that the prognosis became poorer as the WRGs score increased. In contrast to the low‐WRGs group, the levels of GPR68, STAT3, TKTL1, RRM2, and TLR4 expression were higher in the high‐WRGs group, whereas MPC1, SLC16A1, and MTOR expression were the opposite (Figure 2F). In addition, the GSE16011 cohort was also used to verify the prediction value of the Warburg effect signature, where the results of the risk plot were consistent with those of the TCGA training set and the CGGA validation cohort (Figure S4). However, the K‐M curve showed that there was no significant difference in prognosis between high‐WRGs and low‐WRGs groups (*p* = 0.079) (Figure S4).

### 3.3 Identification of the WRGs score as an independent prognostic characteristic

**FIGURE 3:**
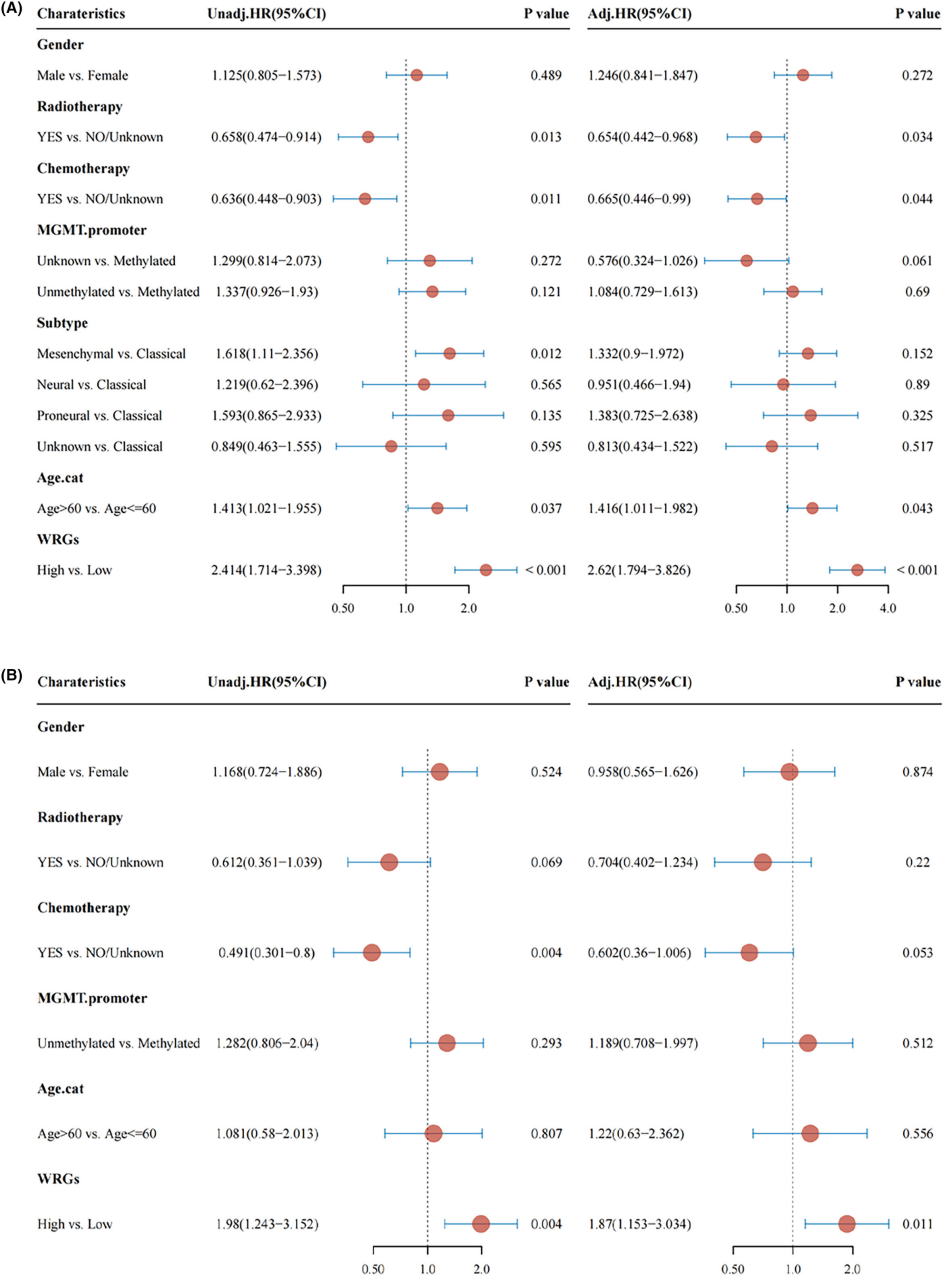
Identification of the WRGs score as an independent prognostic factor. (A) Cox analysis in the TCGA cohort. (B) Cox analysis in the CGGA cohort. Univariate analysis is on the left and Multivariate analysis is on the right.

The role of WRGs score and clinical parameters on the prognosis of GBM was explored by univariate and multivariate Cox regression analyses (Figure 3A). Univariate analysis demonstrated that WRGs score (*p* < 0.001), radiotherapy (*p* = 0.013), chemotherapy (*p* = 0.011), subtype (*p* = 0.012), and age (*p* = 0.037) were significantly related to the prognosis in the GBM cohort of the TCGA. Next, WRGs score (*p* < 0.001), radiotherapy (*p* = 0.034), chemotherapy (*p* = 0.044), and age (*p* = 0.043) were confirmed to independently influence the prognosis of GBM using multivariate analysis. Therefore, the WRGs score calculated by the TCGA GBM cohort was the independent prognostic indicator with the greatest effect on the prognosis of GBM. Moreover, the WRGs score was identified as an independent prognostic characteristic for GBM in the CGGA cohort (*p* = 0.011) (Figure 3B) and the GSE16011 cohort (*p* = 0.037) (Figure S4).

### 3.4 Constructing and validating the nomogram

**FIGURE 4:**
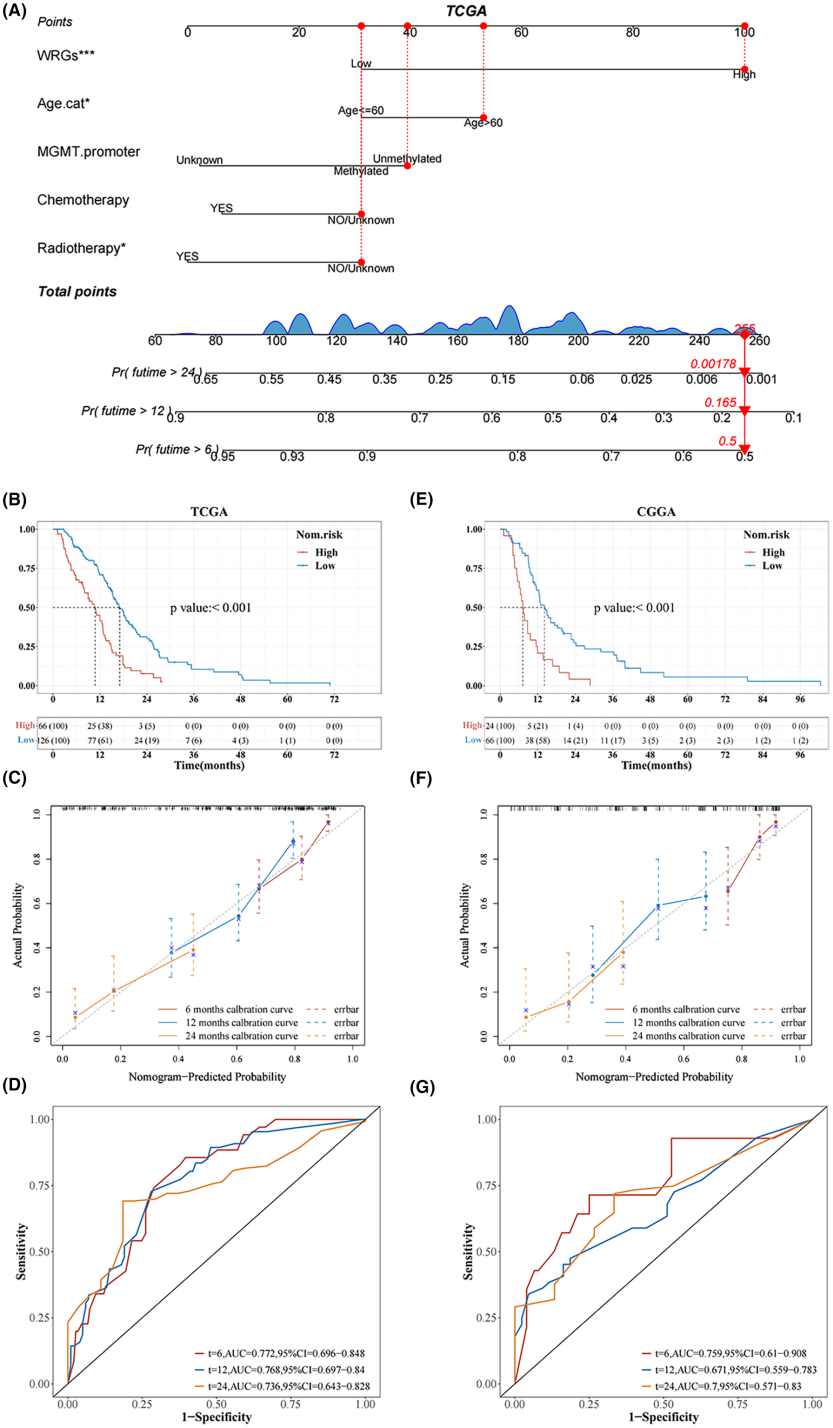
Construction and validation of the nomogram. (A) Nomogram integrating the WRGs score and clinical parameters. (B) Overall survival (OS) comparison between high‐ and low‐WRGs groups according to a nomogram in the TCGA cohort (*p* < 0.01). (C and D) Calibration curve, and area under curve (AUC) of the nomogram at 6, 12, and 24 months of survival in the TCGA cohort. (E) OS comparison between high‐ and low‐WRGs groups according to a nomogram in the CGGA cohort (*p* < 0.01). (F and G) Calibration curve, and AUC of the nomogram at 6, 12, and 24 months of survival in the CGGA cohort.

A predictive nomogram was constructed to clinically predict the probability of 6‐, 12‐, and 24‐month survival in GBM patients. To construct the nomogram, five independent prognostic indicators (WRGs score, age, radiotherapy, chemotherapy, and MGMT promoter) were included, and the results showed that WRGs score had the highest effect on the prognosis of GBM among these factors (Figure 4A). Based on the nomogram, each factor was assigned a score, and then, the sum for every patient with GBM was estimated. According to the scores, the probability of 6‐, 12‐, and 24‐month survival for GBM patients was estimated. The accuracy and sensitivity of the nomogram were validated by the calibration curve, area under curve (AUC) value, and C‐index. The K‐M survival curve demonstrated that the prognosis of the low‐WRGs group was dramatically better in contrast to the high‐WRGs group in the TCGA training cohort (*p* < 0.001) (Figure 4B). The calibration curve revealed that actual observations and the predictions of the nomogram were consistent (Figure 4C). ROC analysis indicated that the nomogram had the ability to accurately predict the OS of patients at 6, 12, and 24 months, with AUC values of 0.772, 0.768, and 0.736, respectively (Figure 4D). The C‐index after bootstrap‐resampling was 0.633. Similar results were identified in the CGGA validation cohort, where the K‐M curve revealed patients in the high‐WRGs group with a much poorer prognosis as compared to the low‐WRGs group (*p* < 0.001) (Figure 4E). The calibration curve indicated excellent consistency between the predictions and actual observations (Figure 4F). The C‐index after bootstrap‐resampling was 0.696. The AUC values indicated the nomogram had excellent predictive ability in the CGGA validation cohort (Figure 4G). The GSE16011 cohort was used to re‐validate the accuracy of the nomogram to predict the OS of GBM patients at 6 months (Figure S4). The calibration curve demonstrated consistency between the predictions and actual observations. The ROC curve demonstrated the nomogram had the ability to accurately predict the OS at 6 months.

### 3.5 Immune infiltration, TMB, and MSI analysis

**FIGURE 5:**
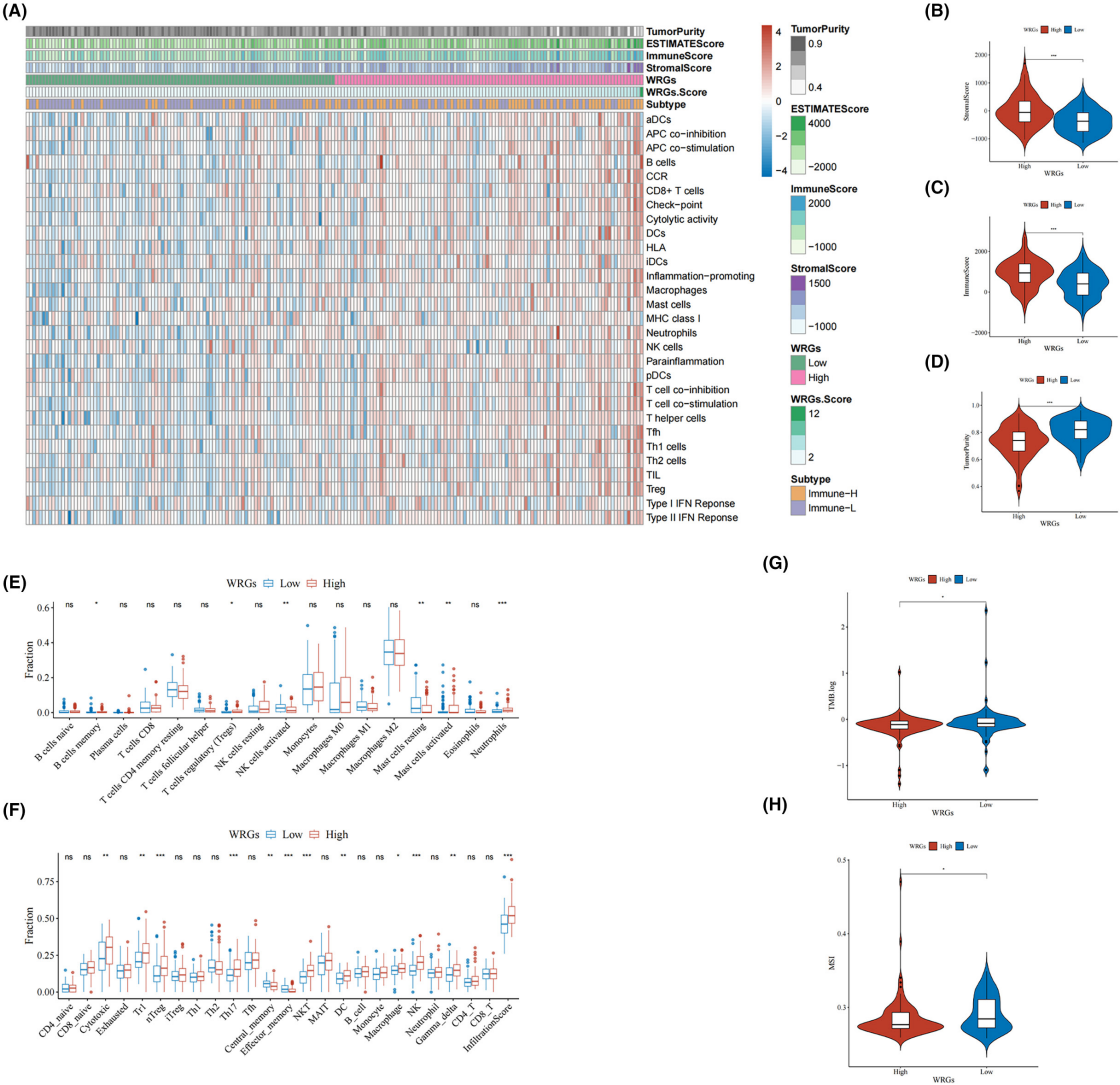
Immune infiltration, tumor mutation burden (TMB), and microsatellite instability (MSI) in the TCGA cohort. (A) Heatmap for immune infiltration between low‐ and high‐WRGs groups. (B–D) Comparison of the stromal score, immune score, and tumor purity between the low‐ and high‐WRGs groups. (E and F) Comparison of immune cell abundance in the low‐ and high‐WRGs groups. (G and H) Comparison of TMB and MSI in low‐ and high‐WRGs groups. *p < 0.05, **p < 0.01, ***p < 0.001.

Based on previous studies, we hypothesized that the Warburg effect might influence the prognosis of GBM by interfering with the immune microenvironment. Thus, the ssGSEA, ESTIMATE, CIBERSORTx, and ImmuCellAI were utilized to assess the differences in immune infiltration between two groups. The results of the ssGSEA revealed the correlation between 29 immune‐related components and WRGs score, with immune‐related components being more activated in the high‐WRGs cluster (Figure S1). Based on this analysis, GBM patients were divided into two groups by hierarchical clustering: high immune infiltration (Immune‐H) and low immune infiltration (Immune‐I). The WRGs score in the immune‐H group dramatically increased as compared to the immune‐I group (Figure S1). ESTIMATE assessed tumor purity and immune microenvironment scores between the low‐ and high‐WRGs groups (Figure 5A). The results revealed that stromal and immune scores were significantly higher in the high‐WRGs group (Figure 5B,C), whereas tumor purity showed an opposite tendency (Figure 5D). The abundance of immune cells was shown by CIBERSORTx analysis (Figure 5E). Immunosuppressive cells, including regular T cells (Tregs), neutrophils, and mast cells activated, were greatly abundant in the high‐WRGs group, whereas immune‐defense cells, such as nature killer cells activated, were highly enriched in the low‐WRGs group. Additionally, ImmuCellAI was utilized to assess the immune cell infiltration in GBM patients, which revealed that the infiltration score of the low‐WRGs group was considerably lower than that of the high‐WRGs group (Figure 5F). The validation in the CGGA cohort (Figure S2) and the GSE16011 cohort (Figure S5) employed the same methodology as above, and the results were in accordance with the training cohort. The results demonstrated that stromal and immune scores were significantly higher in the high‐WRGs group, whereas tumor purity was significantly higher in the low‐WRGs group. Moreover, the difference in sensitivity to immunotherapy between the two groups was also assessed. The results demonstrated that the TMB and MSI in the high‐WRGs group were dramatically lower in comparison with those in the low‐WRGs group (Figure 5G,H), demonstrating that immunotherapy was more helpful for low‐WRGs GBM patients.

### 3.6 Evaluation of drug sensitivity targeting Warburg effect‐related genes

**FIGURE 6:**
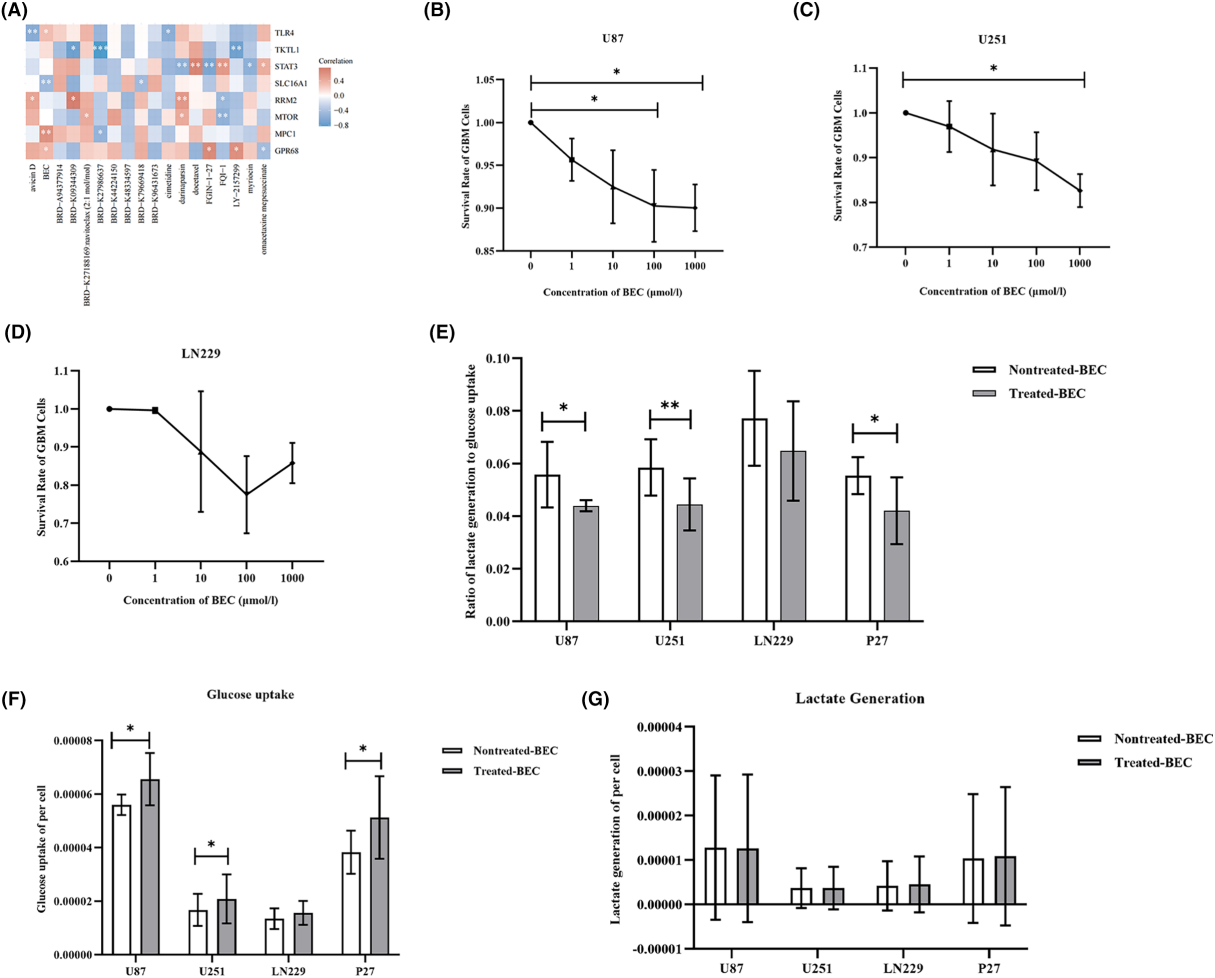
Identification and validation of sensitive drugs targeting Warburg effect‐related genes. (A) Sensitive drugs target eight Warburg effect‐related genes. (B–D) Survival rate of U87, UN251, and LN229 GBM cells after treatment with different concentrations of BEC. (E) Effect of BEC on glucose uptake and lactate production in U87, U251, LN229, and P27 GBM cell lines. (F) Effect of BEC on the glucose uptake in U87, U251, LN229, and P27 GBM cell lines. (G) Effect of BEC on the lactate generation in U87, U251, LN229, and P27 GBM cell lines. Glucose uptake and lactate generation assays were performed five times, respectively. The Wilcoxon test was used for data analyses. *p < 0.05, **p < 0.01.

By statistically comparing the expression of Warburg effect signature related antibodies in low‐ and high‐grade gliomas (HGG) on the Human Protein Atlas website, we discovered that the high expression of Warburg effect signature in HGG was highly expressed in the high‐WRGs group, providing a basis for the treatment of GBM by targeting Warburg effect signature. To identify drugs that were sensitive against Warburg effect signature, drug sensitivity analysis was performed using the CTRP and CCLE databases. The correlation analysis between the dose–response AUC of drugs and the gene expression levels was performed to explore the sensitive drugs of the Warburg effect‐related genes (Figure 6A). The results revealed that BEC was the drug correlating with the most genes in the Warburg effect signature. The dose–response AUC of BEC was positively correlated with the expression levels of TLR4, GPR68, and MPC1 and negatively correlated with the expression level of SLC16A1, suggesting that BEC was more toxic to cells with low levels of MPC1, TLR4, and GPR68 expression and high level of SLC16A1 expression. Moreover, our study revealed high expression levels of TLR4 and GPR68 and low expression levels of SLC16A1 and MPC1 in GBM patients with poor prognosis (Figure 2C). We indicated that BEC may interfere with the Warburg effect by highly targeting MPC1 in GBM patients with poor prognosis. Next, the ability of BEC to inhibit the GBM cells growth was evaluated by the CCK‐8 assays, and the results revealed that BEC could significantly inhibit the growth of U87 GBM cells growth in vitro when its concentration reached 100 μmol/L (Figure 6B). The survival of both U87 (Figure 6B) and U251 (Figure 6C) GBM cells could be inhibited in vitro when the concentration of BEC reached 1000 μmol/L but not the LN229 cell line (Figure 6D). Figure S3 demonstrated that BEC significantly inhibited the growth of the P27 cell line when its concentration reached 100 μmol/L. Similar to the U87 and U251 cell lines, the P27 cell line showed less than 50% growth inhibition even at a BEC concentration of 1000 μmol/L. In addition, the results also revealed that BEC dose‐dependently inhibited the growth of U87, U251, and P27, but not in LN229 cells. Figure 6E showed that the ratio of lactate generation to glucose uptake was significantly lower in U87, U251, and P27 GBM cell lines. The glucose uptake assay demonstrated that glucose consumption in BEC‐treated U87, U251, and P27 cells was significantly higher than that in the untreated group (Figure 6F). However, the lactate generation assay illustrated that there was no significant change between the treated‐BEC and nontreated‐BEC groups (Figure 6G). The results suggested that BEC can interfere with the metabolism of GBM cell lines.

## 4 DISCUSSION

GBM is a glioma with a poor prognosis, drug resistance, and a high incidence of relapse.^30^, ^31^ The Warburg effect is a metabolic phenotype of tumor cells,^6^ which is the main cause of acidic TME and can promote tumor metastasis, immune evasion, and drug resistance.^32^, ^33^ The present study aimed to categorize patients at risk and predict their prognosis for GBM in order to support clinical treatments based on the Warburg effect. In our study, we developed a prognostic risk score model based on Warburg effect‐related genes, and the role of the Warburg effect signature in the immune microenvironment and the prediction of the prognosis of GBM were investigated. In addition, novel drugs that target Warburg effect‐related genes were also explored.

In our study, the WRGs score was the factor that most significantly affected the prognosis of GBM, although other factors such as surgery,^34^ radiotherapy,^35^ and age^36^ showed a more significant effect on the prognosis in previous studies. The possible reason is that our model reclassified the GBM cohort in the TCGA database following the most recent diagnostic criteria for GBM in WHO CNS5. In WHO CNS5, the clinical definition of GBM is updated by the inclusion of molecular parameters such as TERT promoter mutation, EGFR gene amplification, and +7/−10 copy number change.^27^ This indicated that, compared to models from previous studies, our model was more suitable for the current clinical prediction of the prognosis of GBM. However, the prediction accuracy of the model remains to be further confirmed in clinical practice.

Our prognostic model showed low levels of MPC1 expression and high levels of STAT3, RRM2, and TLR4 expression, suggesting a poor prognosis for GBM patients, which was in agreement with previous studies.^37^, ^38^, ^39^, ^40^, ^41^, ^42^ However, our study differed from previous studies in several aspects. A previous study has shown that GBM patients with activated PI3K/Akt/mTOR pathways have a poor prognosis.^43^ In this study, the expression of MTOR was found to be low in patients with poor prognosis. It may be because MTOR in our study referred to transcriptome data, whereas the previous studies referred to protein expression levels. This can be the possible outcome of an inconsistency between the transcription level of a gene and its protein level. Our study also identified many genes that had not previously been linked to the prognosis of GBM. The findings revealed that high levels of GPR68 and TKTL1 expression and a low level of SLC16A1 expression suggested a poor prognosis for GBM. Previous studies have shown that GPR68 promotes connections between tumor cells and cancer‐associated fibroblasts, which in turn can contribute to carcinogenesis.^44^ It also promotes tumor growth by maintaining macrophages in the M2 state and inhibiting the infiltration of T‐cell.^44^ The high level of GPR68 expression possibly indicates the short survival rate of tumors. High expression of TKTL1 has been correlated with poor prognosis in several tumors.^45^ SLC16A1 was shown to be expressed at higher levels in GBM in comparison with LGG.^46^, ^47^ However, the influence of SLC16A1 expression level on the prognosis of GBM remains inconclusive. Therefore, our research was the first to report the effect of expression levels of GPR68, TKTL1, and SLC16A1 on the prognosis of GBM, and the specific effects of these genes in GBM need to be further confirmed.

Analysis of immune infiltration revealed that the abundance of immune‐suppressive cells, such as Tregs and neutrophils, was considerably higher in the high‐WRGs group as compared to the low‐WRGs group, indicating that the Warburg effect may be one of the reasons for immunosuppression in GBM. A possible mechanism is that the accumulation of lactate by the Warburg effect inside the cell might damage the cellular compartment and halt metabolic processes. Excess lactate must be transported from the cell into the TME to prevent intracellular acidification, which results in the acidification of TME.^48^ Acidic TME can induce the invasion of immune cells that have immunosuppressive effects, such as Tregs, M2 macrophages, and N2 neutrophils.^49^ Tregs are currently considered to be the major regulators of immunosuppression in the TME of glioma, and the induction of their activity has contributed to the progression and poor prognosis of glioma.^23^, ^50^ Tumor‐infiltrating neutrophils can inhibit T cells from attacking tumors, and a high percentage of neutrophils is correlated with a short survival time for GBM patients.^51^, ^52^ In light of the aforementioned pathways, the Warburg effect may result in the immunosuppression of GBM and have an impact on the prognosis.

Targeting the Warburg effect is currently considered a promising cancer therapy strategy,^53^ and drugs that target the metabolic enzymes involved in aerobic glycolysis have been explored in GBM.^54^, ^55^ This is the first study to target Warburg effect‐related genes for drug sensitivity and obtain the drug BEC, to which MPC1 is highly sensitive. Our results showed that low expression of MPC1 exhibited high sensitivity to BEC. Downregulation of MPC1 expression has been related to poor prognosis and temozolomide resistance in GBM.^42^ Previous studies have shown that inhibitors of Chicken ovalbumin upstream promoter transcription factor II (COUP‐TFII) can inhibit the growth of GBM by targeting MPC1.^42^, ^56^ According to the CCK‐8 assay, high concentrations of BEC had a growth‐inhibiting effect on GBM cells that did not exceed 50% in vitro. One possible reason for this might be that the absence of an immune microenvironment in our study may have affected the action of BEC since BEC is a known immune‐activating drug.^57^ In addition, the results showed that BEC can interfere with the metabolism of GBM cell lines, which may potentially be one of the mechanisms by which BEC could inhibit the growth of GBM cells. It is, therefore, necessary to further explore the effect of BEC in inhibiting the progression of GBM by targeting Warburg effect‐related genes to provide potential effective drugs for GBM treatment.

There were a few limitations to our research. The CGGA validation set for this study was obtained after excluding LGG and IDH mutant GBM following the fourth edition of the WHO CNS in 2016, which did not include newly upgraded GBM patients in the WHO CNS5 in 2021. Secondly, the prognostic model of Warburg effect‐related genes has not yet been used in clinical practice, which requires further validation in prospective studies and clinical trials.

In conclusion, a trustworthy Warburg effect‐related genes risk scoring model was identified. This model has a significantly excellent predictive value for the prognosis of GBM patients, suggests their immunotherapy response, and identifies new GBM therapeutic targets. In addition, a promising prognostic nomogram was built using a combination of the WRGs score and clinical characteristics to provide an individual prediction of OS and facilitate the selection of effective treatment strategies in the clinic.

## AUTHOR CONTRIBUTIONS

**Rong Zhang:** Conceptualization (equal); data curation (equal); formal analysis (equal); investigation (equal); methodology (equal); visualization (equal); writing – original draft (equal); writing – review and editing (equal). **Can Wang:** Conceptualization (equal); data curation (equal); formal analysis (equal); methodology (equal); software (equal); validation (equal); visualization (equal); writing – review and editing (equal). **Xiaohong Zheng:** Formal analysis (equal); investigation (equal); writing – review and editing (equal). **Shenglan Li:** Data curation (equal); project administration (equal); software (equal). **Weichunbai Zhang:** Data curation (equal); software (equal). **Zhuang Kang:** Data curation (equal). **Shuo Yin:** Data curation (equal). **Jinyi Chen:** Data curation (equal). **Feng Chen:** Data curation (equal); methodology (equal); supervision (equal). **Wenbin Li:** Conceptualization (equal); data curation (equal); funding acquisition (equal); methodology (equal); project administration (equal); supervision (equal); writing – review and editing (equal).

## FUNDING INFORMATION

This study received financial support from the Beijing Advanced Innovation Center for Big Data‐based Precision Medicine, Capital Medical University, Beijing, 100,069, China (2021YFF0901404), the National Natural Science Foundation of China (No.81972338), National Science and Technology Major Project of China (No.2016ZX09101017), Clinical Major Specialty Projects of Beijing, and the Talent Introduction Foundation of Tiantan Hospital (RCYJ‐2020‐2025‐LWB).

## CONFLICT OF INTEREST STATEMENT

The authors declare no conflicts of interest.

## ETHICS STATEMENT

This study was approved by the Beijing Tiantan Hospital of Capital Medical University, Beijing, China. The P27 cell line from passage 27 of primary GBM cells was sought after ethical approval and patient consent.

## REGISTRY AND THE REGISTRATION NO. OF THE STUDY/TRIAL

KY 2021‐153‐03.

Approval of the research protocol by an Institutional Reviewer Board: N/A.

Animal Studies: N/A.

## CONSENT

Written informed consent was obtained from all the patients before inclusion in the study.

## Supporting information


Figure S1:

Figure S2:

Figure S3:

Figure S4:

Figure S5:

Table S1:

## Data Availability

All data in this study are included in the main or supplementary materials. The raw data are available from the corresponding author upon request.
