# Supplementary figures and images for: Warburg effect‐related risk scoring model to assess clinical significance and immunity characteristics of glioblastoma
Source: Cancer Med. 2023 Oct 21;12(21):20639–54. doi: 10.1002/cam4.6627 (PMC10660605; doi:10.1002/cam4.6627)

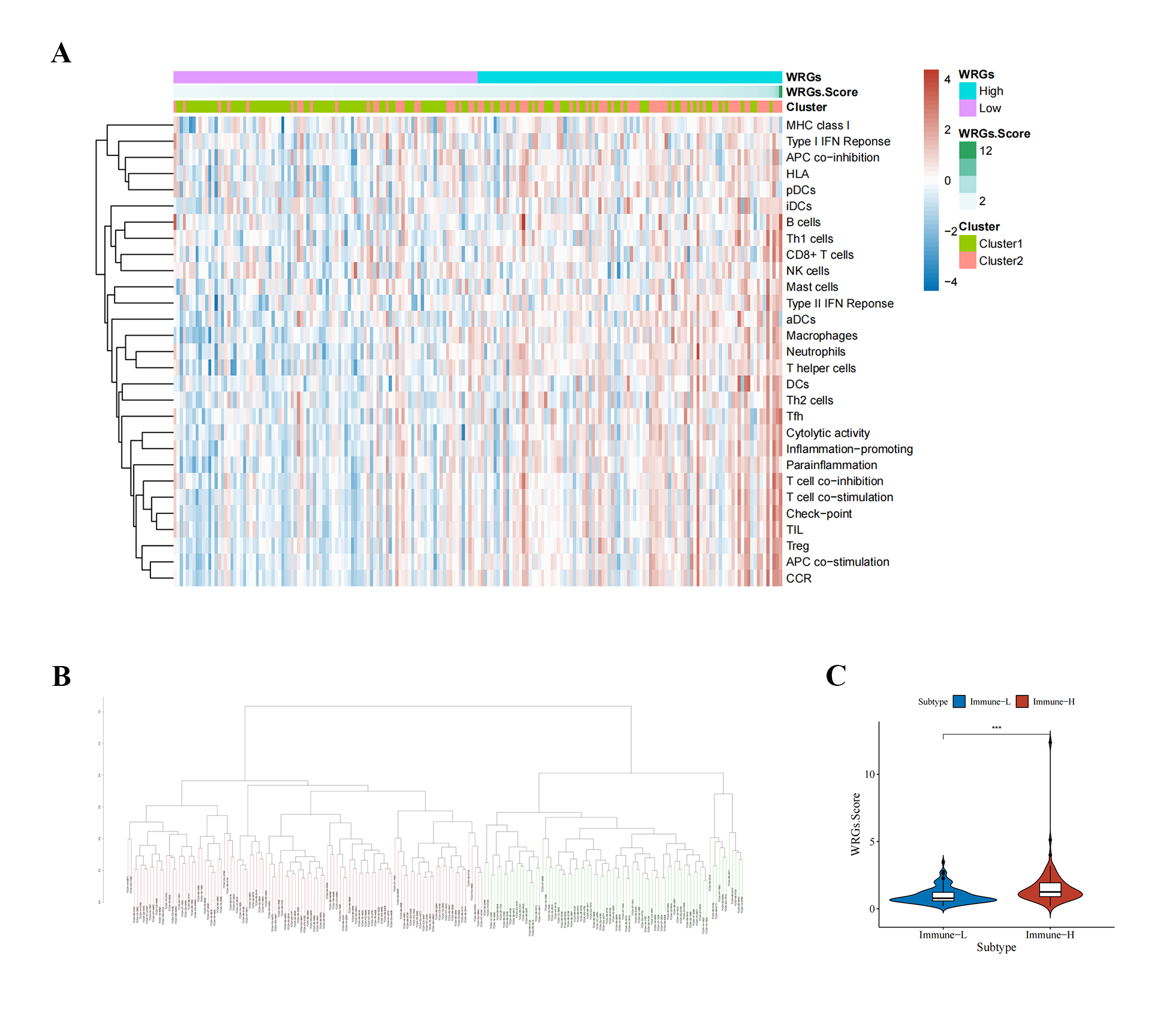

Supplement: Supplementary file 1 — Figure S1: [file CAM4-12-20639-s006.tif]

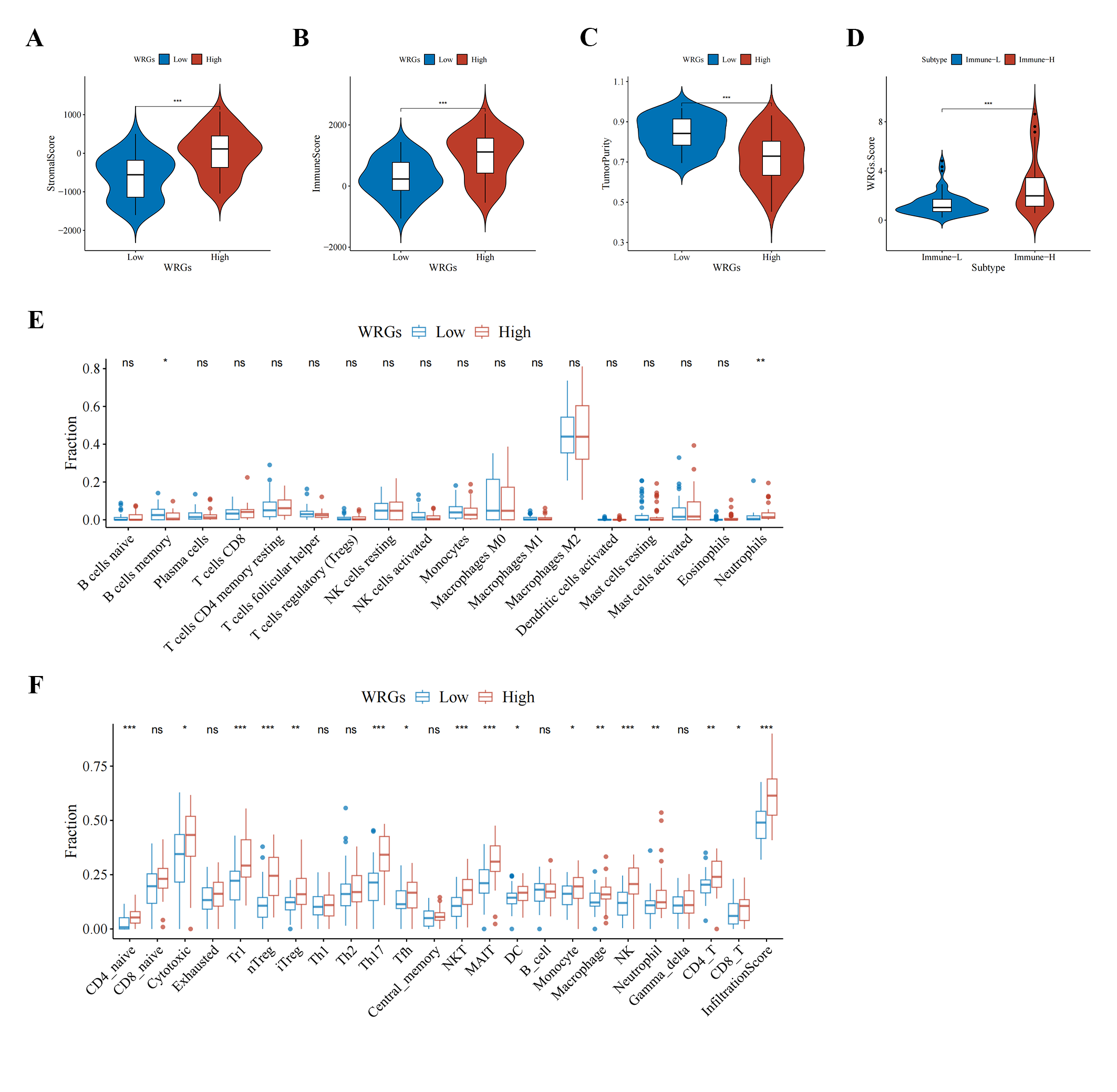

Supplement: Supplementary file 2 — Figure S2: [file CAM4-12-20639-s003.tif]

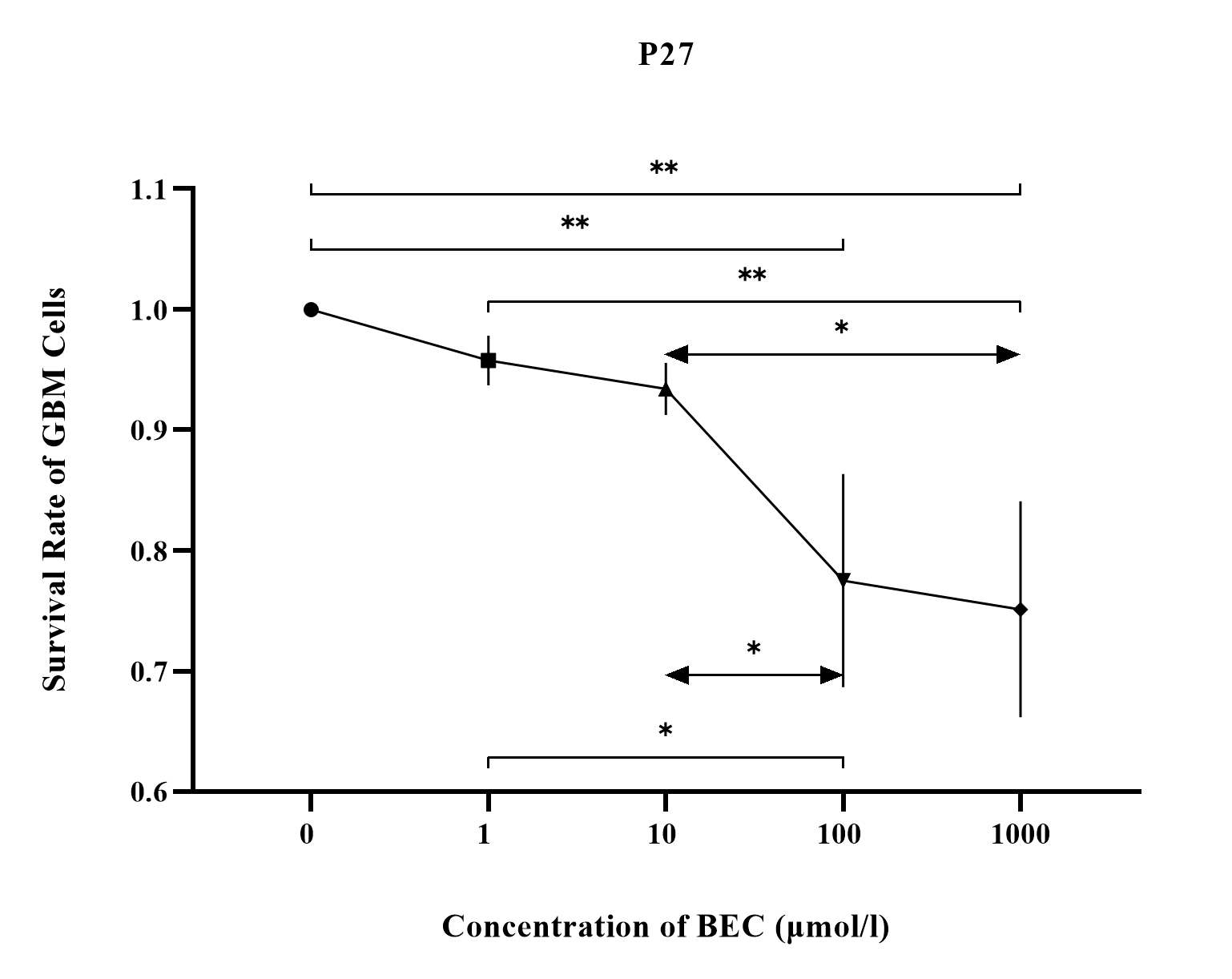

Supplement: Supplementary file 3 — Figure S3: [file CAM4-12-20639-s001.tif]

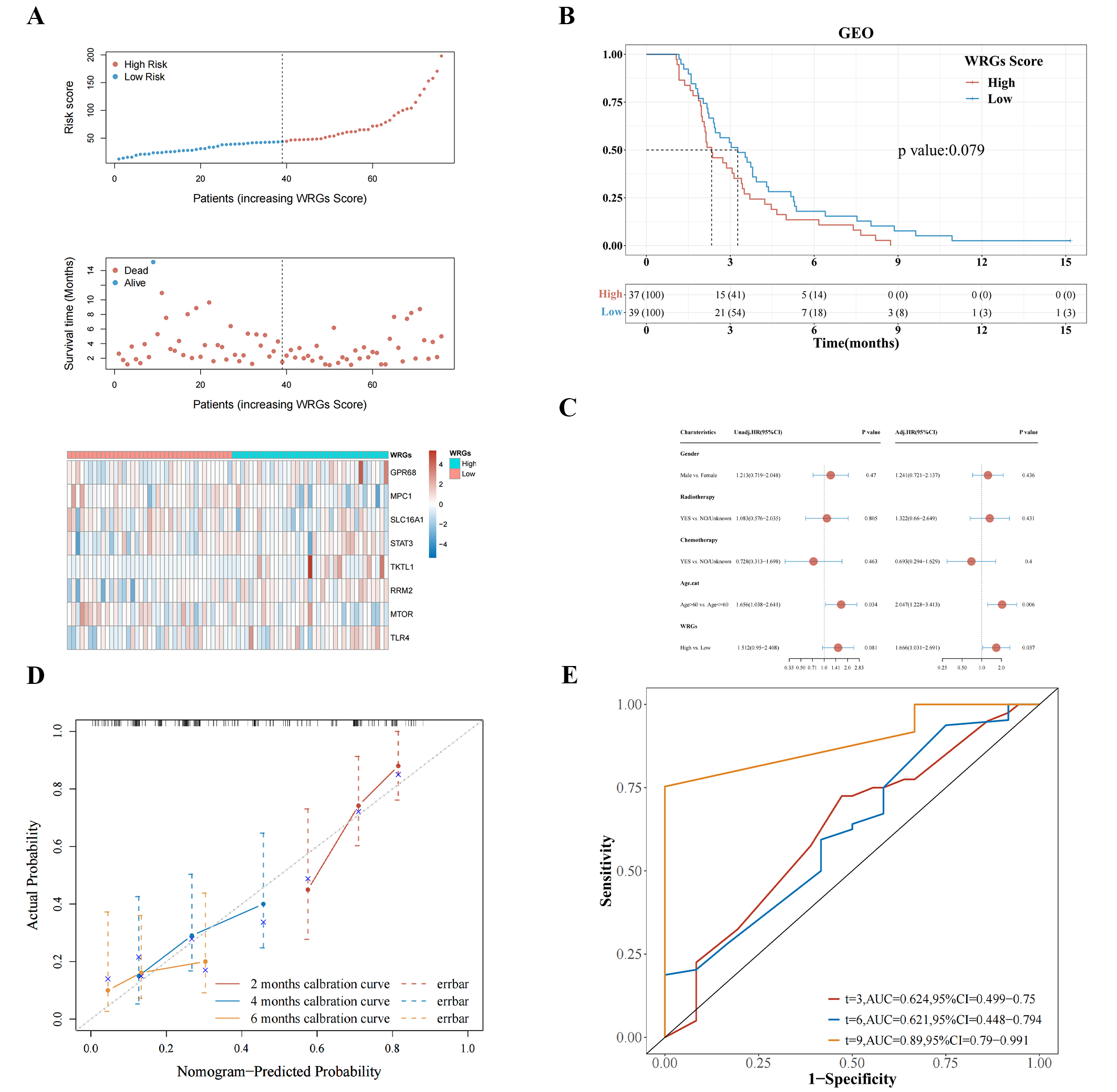

Supplement: Supplementary file 4 — Figure S4: [file CAM4-12-20639-s005.tif]

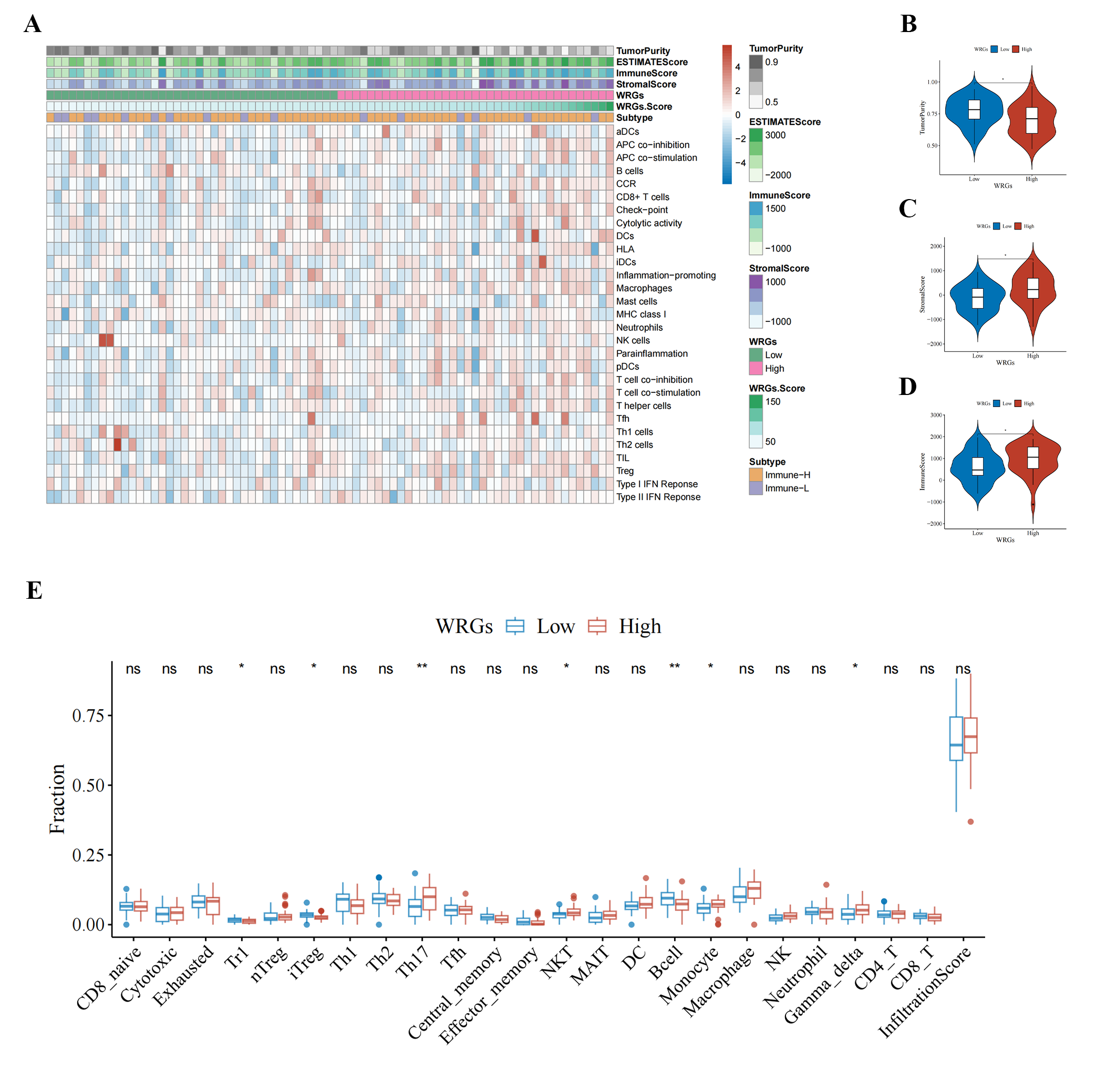

Supplement: Supplementary file 5 — Figure S5: [file CAM4-12-20639-s002.tif]
