# Supplementary material for: Warburg effect‐related risk scoring model to assess clinical significance and immunity characteristics of glioblastoma
Source: Cancer Med. 2023 Oct 21;12(21):20639–54. doi: 10.1002/cam4.6627 (PMC10660605; doi:10.1002/cam4.6627)
Supplement: Supplementary file 6 — Table S1: [file CAM4-12-20639-s004.docx]

**Table S1: Literature sources of Warburg effect-related genes**

| Genes | Full name | Reference |
| --- | --- | --- |
| HIF1A | hypoxia inducible factor 1 subunit alpha | (1) |
| SLC5A12 | solute carrier family 5 member 12 | (1) |
| SLC16A1 | solute carrier family 16 member 1 | (1) |
| HCAR1 | hydroxycarboxylic acid receptor 1 | (1) |
| SLC9A1 | solute carrier family 9 member A1 | (2) |
| SLC16A4 | solute carrier family 16 member 4 | (3) |
| SLC5A4 | solute carrier family 5 member 4 | (3) |
| SLC16A2 | solute carrier family 16 member 2 | (3) |
| SLC16A6 | solute carrier family 16 member 6 | (3) |
| SLC16A7 | solute carrier family 16 member 7 | (3) |
| SLC16A3 | solute carrier family 16 member 3 | (3) |
| SLC16A8 | solute carrier family 16 member 8 | (3) |
| KRT38 | keratin 38 | (3) |
| MPC1 | mitochondrial pyruvate carrier 1 | (3) |
| LAMP2 | lysosomal associated membrane protein 2 | (4) |
| MTOR | mechanistic target of rapamycin kinase | (4) |
| CA9 | carbonic anhydrase 9 | (4) |
| NFKB1 | nuclear factor kappa B subunit 1 | (5) |
| CCL20 | C-C motif chemokine ligand 20 | (5) |
| CXCL8 | C-X-C motif chemokine ligand 8 | (5) |
| CXCL2 | C-X-C motif chemokine ligand 2 | (5) |
| CXCL10 | C-X-C motif chemokine ligand 10 | (5) |
| LDHD | lactate dehydrogenase D | (5) |
| LDHAL6B | lactate dehydrogenase A like 6B | (5) |
| PKLR | pyruvate kinase L/R | (6) |
| PKM | pyruvate kinase M1/2 | (6) |
| IDH1 | isocitrate dehydrogenase (NADP (+)) 1 | (7) |
| AKT1 | AKT serine/threonine kinase 1 | (8) |
| RPE | ribulose-5-phosphate-3-epimerase | (8) |
| HK1 | hexokinase 1 | (8) |
| GPI | glucose-6-phosphate isomerase | (8) |
| G6PD | glucose-6-phosphate dehydrogenase | (8) |
| FGGY | FGGY carbohydrate kinase domain containing | (8) |
| XYLB | xylulokinase | (8) |
| ALDH2 | aldehyde dehydrogenase 2 family member | (8) |
| ALDH3A2 | aldehyde dehydrogenase 3 family member A2 | (8) |
| ALDH1A1 | aldehyde dehydrogenase 1 family member A1 | (8) |
| ADH1C | alcohol dehydrogenase 1C (class I), gamma polypeptide | (8) |
| TKT | transketolase | (8) |
| TKTL1 | transketolase like 1 | (8) |
| TALDO1 | transaldolase 1 | (8) |
| PFKM | phosphofructokinase, muscle | (8) |
| TPI1 | triosephosphate isomerase 1 | (8) |
| PGK2 | phosphoglycerate kinase 2 | (8) |
| OTOR | otoraplin | (9) |
| DLD | dihydrolipoamide dehydrogenase | (10) |
| MT3 | metallothionein 3 | (10) |
| TP53 | tumor protein p53 | (11) |
| STAT3 | signal transducer and activator of transcription 3 | (11) |
| MYC | MYC proto-oncogene, bHLH transcription factor | (12) |
| LDHA | lactate dehydrogenase A | (12) |
| PDK1 | pyruvate dehydrogenase kinase 1 | (13) |
| HK2 | hexokinase 2 | (14) |
| CALCA | calcitonin related polypeptide alpha | (15) |
| UROD | uroporphyrinogen decarboxylase | (15) |
| PIR | pirin | (16) |
| VEGF | vascular endothelial growth factor | (16) |
| NFE2L2 | NFE2 like bZIP transcription factor 2 | (16) |
| HTR2A | 5-hydroxytryptamine receptor 2A | (17) |
| HTR2C | 5-hydroxytryptamine receptor 2C | (17) |
| HTR1B | 5-hydroxytryptamine receptor 1B | (17) |
| SLC5A8 | solute carrier family 5 member 8 | (17) |
| GPR4 | G protein-coupled receptor 4 | (17) |
| TBX1 | T-box transcription factor 1 | (17) |
| GPR65 | G protein-coupled receptor 65 | (17) |
| GPR68 | G protein-coupled receptor 68 | (17) |
| GPR132 | G protein-coupled receptor 132 | (17) |
| BSG | basigin (Ok blood group) | (17) |
| PEA15 | proliferation and apoptosis adaptor protein 15 | (17) |
| ROMO1 | reactive oxygen species modulator 1 | (18) |
| TLR4 | toll like receptor 4 | (18) |
| TGFB1 | transforming growth factor beta 1 | (18) |
| NDRG3 | NDRG family member 3 | (19) |
| LDHB | lactate dehydrogenase B | (20) |
| MPC2 | mitochondrial pyruvate carrier 2 | (21) |
| ABCC1 | ATP binding cassette subfamily C member 1 | (22) |
| ATF3 | activating transcription factor 3 | (23) |
| AURKA | aurora kinase A | (24) |
| BID | BH3 interacting domain death agonist | (25) |
| DUSP1 | dual specificity phosphatase 1 | (26) |
| HELLS | helicase, lymphoid specific | (27) |
| NFS1 | NFS1 cysteine desulfurase | (28) |
| RRM2 | ribonucleotide reductase regulatory subunit M2 | (29) |
| TXNIP | thioredoxin interacting protein | (30) |
| VLDLR | very low density lipoprotein receptor | (31) |
| ZFP69B | ZFP69 zinc finger protein B | (32) |

**Reference**

1. Wang JX, Choi SYC, Niu X, Kang N, Xue H, Killam J, et al. Lactic Acid and an Acidic Tumor Microenvironment suppress Anticancer Immunity. International journal of molecular sciences. 2020;21(21).

2. Kraut JA, Madias NE. Metabolic acidosis: pathophysiology, diagnosis and management. Nature reviews Nephrology. 2010;6(5):274-85.

3. Halestrap AP. Monocarboxylic acid transport. Comprehensive Physiology. 2013;3(4):1611-43.

4. Ibrahim-Hashim A, Estrella V. Acidosis and cancer: from mechanism to neutralization. Cancer metastasis reviews. 2019;38(1-2):149-55.

5. Bourrie BC, Willing BP, Cotter PD. The Microbiota and Health Promoting Characteristics of the Fermented Beverage Kefir. Frontiers in microbiology. 2016;7:647.

6. Akins NS, Nielson TC, Le HV. Inhibition of Glycolysis and Glutaminolysis: An Emerging Drug Discovery Approach to Combat Cancer. Current topics in medicinal chemistry. 2018;18(6):494-504.

7. Ikon N, Ryan RO. Barth Syndrome: Connecting Cardiolipin to Cardiomyopathy. Lipids. 2017;52(2):99-108.

8. Abdel-Rahman MA, Tashiro Y, Sonomoto K. Lactic acid production from lignocellulose-derived sugars using lactic acid bacteria: overview and limits. Journal of biotechnology. 2011;156(4):286-301.

9. de Vos WM, Hugenholtz J. Engineering metabolic highways in Lactococci and other lactic acid bacteria. Trends in biotechnology. 2004;22(2):72-9.

10. Juturu V, Wu JC. Microbial production of lactic acid: the latest development. Critical reviews in biotechnology. 2016;36(6):967-77.

11. Abdel-Rahman MA, Tashiro Y, Sonomoto K. Recent advances in lactic acid production by microbial fermentation processes. Biotechnology advances. 2013;31(6):877-902.

12. Dang CV, Lewis BC, Dolde C, Dang G, Shim H. Oncogenes in tumor metabolism, tumorigenesis, and apoptosis. Journal of bioenergetics and biomembranes. 1997;29(4):345-54.

13. Icard P, Lincet H. A global view of the biochemical pathways involved in the regulation of the metabolism of cancer cells. Biochimica et biophysica acta. 2012;1826(2):423-33.

14. Mathupala SP, Ko YH, Pedersen PL. Hexokinase-2 bound to mitochondria: cancer's stygian link to the "Warburg Effect" and a pivotal target for effective therapy. Seminars in cancer biology. 2009;19(1):17-24.

15. Chandel V, Maru S, Kumar A, Kumar A, Sharma A, Rathi B, et al. Role of monocarboxylate transporters in head and neck squamous cell carcinoma. Life sciences. 2021;279:119709.

16. Martel F, Guedes M, Keating E. Effect of polyphenols on glucose and lactate transport by breast cancer cells. Breast cancer research and treatment. 2016;157(1):1-11.

17. Baltazar F, Afonso J, Costa M, Granja S. Lactate Beyond a Waste Metabolite: Metabolic Affairs and Signaling in Malignancy. Frontiers in oncology. 2020;10:231.

18. Gwangwa MV, Joubert AM, Visagie MH. Crosstalk between the Warburg effect, redox regulation and autophagy induction in tumourigenesis. Cellular & molecular biology letters. 2018;23:20.

19. Marchiq I, Pouysségur J. Hypoxia, cancer metabolism and the therapeutic benefit of targeting lactate/H(+) symporters. Journal of molecular medicine (Berlin, Germany). 2016;94(2):155-71.

20. Ždralević M, Vučetić M, Daher B, Marchiq I, Parks SK, Pouysségur J. Disrupting the 'Warburg effect' re-routes cancer cells to OXPHOS offering a vulnerability point via 'ferroptosis'-induced cell death. Advances in biological regulation. 2018;68:55-63.

21. Ruiz-Iglesias A, Mañes S. The Importance of Mitochondrial Pyruvate Carrier in Cancer Cell Metabolism and Tumorigenesis. Cancers. 2021;13(7).

22. Chen XR, Zhang YG, Wang Q. miR-9-5p Mediates ABCC1 to Elevate the Sensitivity of Glioma Cells to Temozolomide. Frontiers in oncology. 2021;11:661653.

23. Lu S, Wang XZ, He C, Wang L, Liang SP, Wang CC, et al. ATF3 contributes to brucine-triggered glioma cell ferroptosis via promotion of hydrogen peroxide and iron. Acta pharmacologica Sinica. 2021;42(10):1690-702.

24. Du R, Huang C, Liu K, Li X, Dong Z. Targeting AURKA in Cancer: molecular mechanisms and opportunities for Cancer therapy. Molecular cancer. 2021;20(1):15.

25. Neitemeier S, Jelinek A, Laino V, Hoffmann L, Eisenbach I, Eying R, et al. BID links ferroptosis to mitochondrial cell death pathways. Redox biology. 2017;12:558-70.

26. Chen X, Yu C, Kang R, Kroemer G, Tang D. Cellular degradation systems in ferroptosis. Cell death and differentiation. 2021;28(4):1135-48.

27. Hou X, Yang L, Wang K, Zhou Y, Li Q, Kong F, et al. HELLS, a chromatin remodeler is highly expressed in pancreatic cancer and downregulation of it impairs tumor growth and sensitizes to cisplatin by reexpressing the tumor suppressor TGFBR3. Cancer medicine. 2021;10(1):350-64.

28. Alvarez SW, Sviderskiy VO, Terzi EM, Papagiannakopoulos T, Moreira AL, Adams S, et al. NFS1 undergoes positive selection in lung tumours and protects cells from ferroptosis. Nature. 2017;551(7682):639-43.

29. Zhan Y, Jiang L, Jin X, Ying S, Wu Z, Wang L, et al. Inhibiting RRM2 to enhance the anticancer activity of chemotherapy. Biomedicine & pharmacotherapy = Biomedecine & pharmacotherapie. 2021;133:110996.

30. Alhawiti NM, Al Mahri S, Aziz MA, Malik SS, Mohammad S. TXNIP in Metabolic Regulation: Physiological Role and Therapeutic Outlook. Current drug targets. 2017;18(9):1095-103.

31. Boycott KM, MacDonald SK, Parboosingh JS. VLDLR Cerebellar Hypoplasia. In: Adam MP, Mirzaa GM, Pagon RA, Wallace SE, Bean LJH, Gripp KW, et al., editors. GeneReviews(®). Seattle (WA): University of Washington, Seattle

Copyright © 1993-2023, University of Washington, Seattle. GeneReviews is a registered trademark of the University of Washington, Seattle. All rights reserved.; 1993.

32. Zheng X, Wang X, Zheng L, Zhao H, Li W, Wang B, et al. Construction and Analysis of the Tumor-Specific mRNA-miRNA-lncRNA Network in Gastric Cancer. Frontiers in pharmacology. 2020;11:1112.
